# Comparison of Titanium vs. Polycel Total Ossicular Replacement Prosthesis

**Published:** 2016-03

**Authors:** Mohammad Faramarzi, Reza Jahangiri, Sareh Roosta

**Affiliations:** 1 *Otorhinolaryngology Research Centre, Shiraz University of Medical Sciences, Shiraz, Iran.*; 2 *Department of Otorhinolaryngology, Shiraz University of Medical Sciences, Shiraz, Iran.*

**Keywords:** Chronic otitis media, Ossicular reconstruction, Total ossicular replacement prosthesis, Titanium, Polycel.

## Abstract

**Introduction:**

Even though modern technology progresses so rapidly, annals of otology are replete with so many challenging article, which often compare various types of prosthesis. Since there has not been a prospective randomized clinical trial study which compares the hearing result of total ossicular replacement prosthesis made of Titanium with omega connector and Polycel in the literature, we decided to perform a study encompassing this issue.

**Materials and Methods:**

105 patients, who were in the 2nd stage of their operation and who needed total ossicular replacement prosthesis, were included in this prospective single blind randomized clinical trial study. Patients were classified in two groups: titanium Kurz (TTP™ -Vario system, Kurz GmbH, Dusslingen, Germany) with omega connector and Polycel (Sheehy Plastipore Polycel, Medtronic Xomed Inc). The duration of the follow up was 6-12 months. In order to evaluate hearing results, pure tone audiometric in 0.5, 1, 2, and 4 kHz were checked. In addition, speech reception threshold was recorded. A successful surgery was defined as having a postoperative air–bone gap within 20 dB.

**Results:**

We accomplished successful hearing in 64.4% of patients with titanium and 65% of patients with a Polycel prosthesis.Improvement in speech reception threshold was 11.5 dB in the titanium group and 13 dB in the Polycel group. In other words, there was no significant difference between the two groups. In addition, air-bone gap improvement after ossiculoplasty was 11.2 dB in the patients with a titanium prosthesis and 12.4 dB in the Polycel group. In fact, the difference was not significant.

**Conclusion:**

We found that both the titanium and the Polycel prosthesis improve speech reception threshold and air-bone gap closure in a similar manner.

## Introduction

Despite the advances in the field of ear surgery, otologists are still having a challenging problem in treating chronic otitis media (COM).In the presence of infection, ossicles are destroyed by the osteoclast cell. Some authors have revealed that pseudomonas aeruginosa is the most common organism found in COM (1).Unfortunately about 60%-82% of patients, who refer to otology clinics, have ossicular defect (2,3). There are three common types of ossicular reconstruction in COM surgery. Partial ossicular replacement prostheses (PORP) refer to the operation in which the prosthesis extends from a stapes supra-structure to the malleus or tympanic membrane. Total ossicular replacement prostheses (TORP) refers to the operation in which prostheses are placed between the stapes footplate to the malleus or tympanic membrane. Incus interpositional prosthesis, refers to the operation in which the curved incus bone is connected to the stapes capitulum into the malleus (4).

Several prostheses have been made out of several allograft materials. Plastipore is composed of polyethylene, firstly introduced by Shea in 1976 (5). It has numerous pores, which help stabilization of the prostheses because of the middle ear mucosal ingrowths. Titanium, as a biocompatible metal, has for many years been used for orthopedic and craniofacial surgery and the use of titanium in ear surgery began in 1993 in Germany (6,7). Stupp et al conducted the first study on the hearing outcome with titanium ossicular prostheses in 1999 (8). Since then, the use of titanium prostheses has increased. Some advantages of titanium are its lightness, strength, corrosion-resistance, and biocompatibility. Because of its low ferromagneticity, low specific density, high biocompatibility, and lightweight and rigidity it is a good material for ossicular reconstruction (9-12).Polycel is also a popular prosthesis. It is made with thermal-fused polyethylene material that causes little immune response and it is inserted easily in the middle ear. 70-80% of this prosthesis volume is made by multiple pores of about 250 micrometer in diameter (13).There are many retrospective studies in the literature regarding the effect of various types of prostheses on the improvement of hearing (Table.1). 

**Table 1:** Success rates in some retrospective studies after PORP and TORP ossiculoplasty

**Author (year)**	**Duration of Followup**	**Type of prosthesis** **(number)**	**ABG ≤20dB** **in PORP**	**ABG≤20dB** **in TORP**	**Extrusion or displacement rate**	**Type of study**
Berenholz(2013)(14)	3yr	Plastipore (152)	81.2%	60%	0.6%	retrospective
Mardassi(2011)(11)	9 m	Titanium (33)	86.49%	54.55%	5.7%	retrospective
Kim(2010)(13)	1yr	Polycel (136)	40.2%	31.5%	4.4%	retrospective
Roth (2009)(7)	1-5yr	Titanium (55)	85%	77%	1.8%	retrospective
Huttenbrink (2009)(15)	3 wk	Titanium angular clip(22)	100%	----	NM	retrospective
Neudert (2009)(16)	5yr	Titanium angle (10) Autologous incus (27) Titanium clip(29)	75% 74% 66%	----	NM	retrospective
Eleftheriadou(2009)(17	14yr	Plastipore (42)	68.8%	62.5%	4.7%	retrospective
Coffey (2008)(18)	15 m	Titanium (80) HA (25)	81.5% 50%	74.4% 50%	3.8% 8%	retrospective
Redaelli (2008)(19)	18 m	Titanium (26) HA (24)		46.2% 33.3%	0%	retrospective
Truy (2007)(20)	5yr	Titanium (62) HA (106)	72% 63.2%	45.8% 50%	1.78%	retrospective
Schmerber (2006)(21)	20m	Titanium (111)	52%	77%	1.8%	retrospective
Martin (2004)(12)	3m-2.5yr	Titanium (68)	68%	40%	1.5%	retrospective
Mostafa (2013)(3)	12m	Vario Titanium (8) Classic Titanium (8)	83% 75%	---	NM	Prospective randomized comparative
Yung (2010)(22)	2 yr	Titanium (41) HA (46)	83% 84%	75% 56%	16% 4.5%	Prospective, Randomized control
Alaani (2010)(10)	6 m	Kurz titanium (97)	84	75%	2.06%	prospective
Michael (2008)(6)	12 m	Kurz titanium (14)	78%	---	7%	prospective
Siddiq (2007)(28)	6-18m	Kurz titanium (33)	85%	46%	0%	prospective
Recent study (2013)	6-12 m	Titanium (45) Polycel (60)	---	64.4% 65%	4.4% 6.7%	Prospective randomized Single blind

NM= Not Mentioned, yr: Year, m: Month, wk: Week

Since there was no prospective single blind randomized clinical trial study that compares the effect of TORP made of titanium (Kurz Vario) with Ω (Omega) connector and Polycel in the literature, we decided to carry out this study to compare the hearing results between the titanium and Polycel prosthesis. 

## Materials and Methods

From June 2012 to December 2013, 105 patients participated in this prospective randomized single blind study. The research protocol was approved by the Shiraz University Ethics Committee. Written preformed consent was obtained from all patients.The design of this study was approved by the Iranian Registry of Clinical Trials, Primary Registry in the WHO Registry Network, with the acceptation code: IRCT201205049631N1.

All primary and secondary operations were performed by one academic otologist in Dastgheib hospital, affiliated with the Shiraz University of Medical Sciences. This hospital is a tertiary health care center in the south of Iran. As it is routine in our center, the first stage of the surgery to treat COM can be performed by 3 common types of surgery such as tympanoplasty, canal wall up mastoidectomy (CWUM), and CWDM. Indication of operation was based on the type and extension of pathology such as cholesteatoma, granulation tissue, and middle ear tympanosclerotic plaque. All primary surgery was performed with a post-auricular approach and fascia temporalis. It was used as a tympanic membrane graft. Silastic sheet was inserted over the promontory in all cases. Tympanoplasty with the palisade cartilage technique was used in 7 cases of adhesive tympanic membrane. They belong to the tympan- oplasty group.

In the second stage of the operation, all patients who needed TORP were included in this study. The period between the first and second operation was 8 to 17 months. Patients with incomplete post-operative follow up were excluded from this study. The patients were classified randomly into two groups according to the type of prostheses used as a material for ossiculoplasty: titanium Kurz (TTP™ -Vario system, Kurz GmbH, Dusslingen, Germany) with omega connector and Polycel (Sheehy Plastipore Polycel, Medtronic Xomed Inc). For randomization, we used blocked randomization. Unequal number of groups is due to patients’ withdrawal from the study and loss of the availability of the prosthesis.

Preoperative audiometry was performed one week prior to the operation and first postoperative audiometry was performed 3 months after the surgery; the duration of the follow up was 6-12 months. All patients were reevaluated postoperatively during the follow up period, and examination was done by micro-otoscopy. In order to evaluate hearing outcomes, speech reception threshold (SRT), and pure toneaudio- metry (PTA) in 0.5, 1, 2, and 4 kHz were checked. In our center 3000 Hz frequency is usually not evaluated. Consequently, we calculated it as mean of 2 and 4 kHz frequencies. Mean differences in the threshold were calculated for air conduction (AC), bone conduction (BC), and ABG. In this study, success was defined according to the last audiometry (at least 6 months after the operation) in addition to the guidelines delineated by the Committee on Hearing and Equilibrium of the Academy of Otolaryngology-Head and Neck Surgery. It is defined as a successful hearing result,such as cases in which the patient’ssuchsuch as cases in which the patient’s postoperative ABG is 20 dB or less.

The Statistical Package for Social Science, SPSS for Windows, version 16.0 (SPSS Inc., Chicago, III., USA) was used for data analysis.For continuous variables, inde- pendent groups were compared using the t-test or Mann-Whitney test, whereas paired-compared comparison was made using paired t-test or Wilcoxon test. Relationships between categorical variables were assessed with Chi-square test or Fisher’s exact test. Our criterion for statistical significance was set at P<0.05 for all hypotheses testing and was two-tailed.

## Results

The total population in this study included 105 ears of 105 patients with a mean age of 32 years (range from 15-61 years). 60 ears (57%) belong to female patients and 45 ears (43%) to male patients. Ossiculoplasty was performed in the right ear side in 50 patients (48%) and in the left ear side in 55 (52%). All of these patients were operated in the two stages by the first author (M.F.). 

Tympanoplasty technique was performed in first step of surgery in 46 patients (44%), CWDM in 42 (40%), and CWUM in 17 patients (16%). During the first operation, cholesteatoma was detected in 29.5% (n=31) of patients, followed by granulation tissue in 15.2% (n=16), and tympanosclerosis plaque in 32.2% (n=34). Titanium TORP with Omega connector was used in 45 patients and Polycel in 60 patients. We achieved successful hearing in 29 (64.4%) ears using the titanium prosthesis and 39 (65%) ears in the Polycel group (Table. 2). These success rates were not significant ineither groups (P= 0.95).The effect of the previous primary pathology such as cholesteatoma, granulation tissue, and tympanosclerotic plaque on the success rate was evaluated (Table.2). Data analysis show that there was not a significant difference in success rate regarding previous middle ear pathology between the two types of prostheses. 

We also assessed the effect of the type of primary operation on the success rate in the second operation (Table.2).Our results showed that; however, the success rate in tympanoplasty and CWUM patients was higherthan in CWDM patients; but the difference was not statistically significant (P=0.82). 

As Table 3 shows, there was no significant difference in hearing outcomes between the two prostheses (P>0.05).We evaluated the mean ABG gain in different frequencies in the Titanium and Polycel prostheses (Table.4). The difference was not statistically significant (P>0.05).

Sensory neural hearing loss occurred in 3 patients in the Polycel group (5%) and 2 (4.4%) in the titanium group (P≅1). Also during the follow up, overall extrusion rate was slightly higher in the Polycel group (6.7%, n=4) versus the titanium group (4.4%, n=2); but it was not significant statistically (P= 0.70).

**Table 2:** Effect of previous middle ear pathology and the type of primary operation on success rate in each prosthesis

	**Titanium** **(N=45)**	**Polycel** ** (N=60)**	**Total** **(N=105)**	
	**Success rate n(%)**			**P-value**
All ears	29(64.4)	39(65)	68(64.8)	0.95
**previous middle ear pathology in primary surgery**				
Cholesteatoma	10(71.4)	10(58.8)	20(64.5)	0.71
Granulation tissue	3(42.9)	8(88.9)	11(68.8)	0.11
Tympanosclerotic plaque	11(68.8)	12(66.7)	23(67.6)	0.90
Normal	7(70)	13(61.9)	20(64.5)	0.99
**Primary operation**				
Tympanoplasty	11(57.9)	19(70.4)	30(65.2)	0.38
CWUM mastoidectomy	5(100)	7(58.3)	12(70.6)	0.24
CWDM mastoidectomy	13(61.9)	13(61.9)	26(61.9)	0.99

**Table 3:** Preoperative and postoperative hearing results in Titanium and Polycel prostheses

	**Titanium** (N=45)	**Polycel** (N=60)	**P-value**
Preoperative AC§	54.0 ±10.9**	48.8 ±9.5**	0.01
Postoperative AC	40.5 ±12.8	36.7 ±13.0	0.13
Improvement	13.5 ±14.9(12.5)	12.2 ±15.9(11.2)	0.66
Preoperative BC§	18.1 ±7.9*	10.9 ±5.0	0.00
Postoperative BC	15.8±8.6	11.2 ±6.1	0.00
Improvement	2.3±9.1(1.2)	-0.3±6.6(-1.2)	0.10
Preoperative ABG§	36.0±8.10**	37.9±9.17**	0.27
Postoperative ABG	24.7±7.7	25.4±10.7	0.71
Improvement	11.2± 11.9(10)	12.4 ±15.04(11.2)	0.65
Preoperative SRT	54.9 ± 10.9**	50.8 ± 10.1**	0.06
Postoperative SRT	43.3 ± 16.8	37.8 ± 13.8	0.07
Improvement	11.5 ± 17.2(10)	13 ± 16.2(15)	0.66
			
			

Values are Mean ± SD (Median).

§ Frequency of 0.5-3 kHz

* P<0.05 and

** P<0.001 for within group comparison

**Table 4:** Mean ABG gain in different frequencies in Titanium and Polycel prostheses

	**Prosthesis**	**500Hz**	**1000 Hz**	**2000 Hz**	**3000 Hz**	**4000 Hz**
Mean ABGgain	Titanium	10.8 ± 15.5	12.4± 15.3	13.7± 14.3	8± 11.2	7.3± 12.5
	Polycel	14.1 ± 17.2	12.3± 17.1	11.2± 17.6	12.2± 16.2	11.2± 13.9
	P-value	0.46	0.93	0.39	0.15	0.08

## Discussion

Despite recent developments in prosthesis innovations, there are considerable controversies regarding the type of prosthesis that should be used. Also, the functional outcome is variable in the studies (3). In this single blind randomized clinical trial, we found that there was no significant difference in postoperative hearing outcome between newer titanium TORP with Omega connector and older Polycel TORP. We found that titanium had no superiority over the Polycel regarding SRT improvement and ABG closure.

As Table 1 shows, there are variations in the success rate in retrospective studies with TORP (31.5%-77%), similarly to prospective studies (46%-75%). Our success rate in this study is within the range found in literature.

Literature review showed that variation in success rate in ossiculoplasty depends on several factors. Some authors found that partial ossiculoplasty has a high stability in the long term compared to total ossiculoplasty(14,21,23-27). In contrast, others believe that TORP prostheses provide a better outcome (28-30). Other researchers detected that there was no difference between the PORP and TORP groups (8,31-33).

Another factor that affects hearing results is the type of previous operation such as tympanoplasty, CWUM, and CWDM. Some authors concluded that the overall result after tympanoplasty and CWUM was significantly better than in CWDM (14,21,29,34). Berenholz et al confirmed that due to the severity of the primary disease in the CWDM and the absence of the post-canal wall, ossiculoplasty is more difficult. Also, it was found that a movable ear drum and normal middle ear mucosa in these patients is another problem (31). But others reported no significant difference between prosthesis in CWDM versus CWUM surgery (9). However, our results showed that the success rate in tympanoplasty and CWUM was more than in CWDM patients, but the difference was not significant. Therefore, our data is similar to those of Alaani’s study (10).

Ossiculoplasty can be performed in a one or two stage operation. Yung classified the cause of failure in three categories: prosthesis related, surgeon related, and middle ear inflammation and infection (22). Also, Jaryszak et al in their study reported that Pseudomonas aeruginosa forms biofilm on HA and plastipore and makes them susceptible to being displaced compared with titanium (35). In order to obtain a better hearing result, the tympanic membrane should be intact and no sign of infection should be observed (36). Therefore, we preferred to perform ossiculoplasty in the second stage as a routine after healing of the middle ear mucosa and tympanic membrane. But some authors such as Mishiro et al did the operation for some patients in the first stage and others reported no significant difference between primary or secondary operations(10,37).

There are many controversies in literature about the type of prosthesis that should be used. One of the reasons for choosing a certain type of prosthesis is the ease of insertion of the prosthesis. Hydroxyapatite is made from a brittle material that is easily shattered if cut (38). In contrast, Plastipore is easily cut with a sharp knife after measurement. On the other hand, titanium is stronger than Plastipore. Also, in order to better observe the footplate, windows were designed in the head of the prosthesis; therefore, the bottom of the prosthesis and the footplate was visible during the insertion of the prosthesis(21). Eleftheriadou et al in 2009 conducted a study on 42 patients who underwent ossiculoplasty with Plastipore; they were categorized into 3 groups (tympanoplasty, CWDM, CWUM). A good success rate was reported (gain ABG ≤ 20dB) in 65% and low extrusion rate was also seen (4.7%) (17). Measurement of the appropriate length of the prosthesis is key in the post-operative function of the prosthesis. Titanium is designed in two models. One is considered classic and has a fixed length (Kurz) and the other is the Vario model that should be modified intra-operatively to the required length. In a recent prospective randomized comparative study by Mostafa et al, two different types of titanium implants were compared. They found that although Vario type takes more operation time for shaping the prosthesis, there is no significant difference in improving the ABG between both groups (3). In addition, the Kurz Omega Connector as a ball joint helps to easily connect the TORP to the footplate in different positions and various angles between the TORP, tympanic membrane, and the footplate (8). Similar to our study, some authors reported about titanium and hydroxyapatite, and found no significant difference between these prostheses (20,29).

Despite these advantages, post-operation inflammation and infection and prosthesis extrusion are the main disadvantages of this operation using synthetic prosthesis. Eustachian tube dysfunction and inflam- mation of the middle ear are some of the known causes of this complication (38). As shown in table 1, there is a large variation in extrusion rate of prosthesis (0%- 16%).

In a recent study, extrusion occurred in 2 cases (4.4%) of Kurz titanium and in 4 cases (6.7 %) of Polycel. In fact, extrusion rate has a direct relationship to the follow up time. For example, Shinohara et al reported extrusion in 14 of 106 patients in 3 years of follow up and the 3 other patients developed these complications 2 years later (38). Also, Brenski and Isaacson reported that insertion of a cartilage graft between the implant and tympanic membrane can reduce the chance of prosthesis extrusion rate (39).

Also, sensorineural hearing loss is the other rare complication of ossiculoplasty. Berenholz et al reported that this problem can occur in less than 1% of cases and opening of the oval window may be the predisposing factor (31). Siddiq et al reported no sensory neural hearing loss (23). In our study, sensory neural hearing loss occurred in 3 patients of the Polycel group (5 %) and in 2 of the titanium group (4.4%). Sensorineural hearing loss is a rare complication after ossiculoplasty that may be due to trauma to the footplate causing perilymphatic leakage or due to labyrinthitis. The most prevalent complication is the remaining conductive hearing loss that may be due to prosthesis displacement or fixation or resorption of bony prosthesis (40).

The major strength of this research is that all operations were performed by one surgeon. Surgeon experience is an important factor that influences hearing result after ossiculoplasty, as seen in Charlett’s study, which reported a better outcome in groups operated by a senior surgeon (41).

The limitation of this study is its short-term follow up. Time of follow up plays an important role in evaluation the performance of the prosthesis. Berenholz et al conducted a retrospective review about plastipore prosthesis function in short-term (mean 7.5 months) and long-term (4.3 years) follow up and showed that air-bone gap closure of 10 dB or lower was achieved for 44.1% of the TORP patients while 75% of patients were within 20 dB. However, in long term follow up these decreased to 20% for ABG ≤10dB and 60% for ABG ≤20dB (14). Mishiro et al reported a significant difference in the success rate between 6 months and 5 years of follow up in patients with cholesteatoma who were operated in the first stage, but no difference in patients who were operated in two stages and finally recommended that a two stage operation is useful for long term hearing results (37). Truy et al found deterioration in their success rate by 0.17 dB per month (20).

An ideal prosthesis should be available at a reasonable price. The cost of Kurz Vario titanium TORP with omega connector in our country is at least 5 times more expensive than Polycel (500 USD versus 100 USD). Since hearing outcome is similar in both of them, we recommended the more economical one.

## Conclusion

The final conclusion of our research is that there is no difference between Kurz Vario titanium TORP with omega connector and Polycel regarding post-operative hearing outcome. Therefore, selection of each of them depends on the preference of the surgeon regarding its availability and price. Obviously we cannot generalize our results. Consequently, additional multicenter studies with a larger population is certainly warranted to further investigate and describe the long-term hearing results associated with each prostheses.

## References

[1] Nason R, Jung JY, Chole RA (2009). Lipopolysaccharide-induced osteoclastogenesis from mononuclear precursors: a mechanism for osteolysis in chronic otitis. Journal of the Association for Research in Otolaryngology: JARO.

[2] Albera R, Canale A, Piumetto E, Lacilla M, Dagna F (2012). Ossicular chain lesions in cholesteatoma. Acta otorhinolaryngologica Italica: organo ufficiale della Societa italiana di otorinolaringologia e chirurgia cervico-facciale.

[3] Mostafa BE, Fiky LE, Hassan O (2013). Functional results in ossiculoplasty with different titanium prostheses. Egyptian Journal of Ear, Nose, Throat and Allied Sciences.

[4] Kim HH, Wiet RJ (2003). Preferred technique in ossiculoplasty. Operative Techniques in Otolaryn- gology-Head and Neck Surgery.

[5] Shea J (1976). Plastipore total ossicular replacement prosthesis. The Laryngoscope.

[6] Michael P, Fong J, Raut V (2008). Kurz titanium prostheses in paediatric ossiculoplasty--short term results. International journal of pediatric otorhinolaryngology.

[7] Roth JA, Pandit SR, Soma M, Kertesz TR (2009). Ossicular chain reconstruction with a titanium prosthesis. The Journal of laryngology and otology.

[8] Stupp CH, Stupp HF, Grun D (1996). [Replacement of ear ossicles with titanium prostheses]. Laryngo- rhino- otologie.

[9] Mantei T, Chatzimichalis M, Sim JH, Schrepfer T, Vorburger M, Huber AM (2011). Ossiculoplasty with total ossicular replacement prosthesis and Omega Connector: early clinical results and functional measurements. Otology & neurotology.

[10] Alaani A, Raut VV (2010). Kurz titanium prosthesis ossiculoplasty--follow-up statistical analysis of factors affecting one year hearing results. Auris, nasus, larynx.

[11] Mardassi A, Deveze A, Sanjuan M, Mancini J, Parikh B, Elbedeiwy A (2011). Titanium ossicular chain replacement prostheses: prognostic factors and preliminary functional results. European annals of otorhinolaryngology, head and neck diseases.

[12] Martin AD, Harner SG (2004). Ossicular reconstruction with titanium prosthesis. The Laryngoscope.

[13] Kim HC YM, Kang HS, Ahn JH, Chung JW (2010). Factors Influencing Hearing Outcomes after Ossiculoplasty Using Polycell® Prosthesis in Patients with Chronic Otitis Media. Korean J Audiol.

[14] Berenholz LP, Burkey JM, Lippy WH (2013). Short- and long-term results of ossicular reconstruction using partial and total plastipore prostheses. Otology & neurotology.

[15] Huttenbrink KB, Luers JC, Beutner D (2009). Titanium angular clip: a new prosthesis for reconstruction of the long process of the incus. Otology & neurotology.

[16] Neudert M, Zahnert T, Lasurashvili N, Bornitz M, Lavcheva Z, Offergeld C (2009). Partial ossicular reconstruction: comparison of three different prostheses in clinical and experimental studies. Otology & neurotology.

[17] Eleftheriadou A, Chalastras T, Georgopoulos S, Yiotakis J, Manolopoulos L, Iliadis I (2009). Long-term results of plastipore prostheses in reconstruction of the middle ear ossicular chain. ORL.

[18] Coffey CS, Lee FS, Lambert PR (2008). Titanium versus nontitanium prostheses in ossiculoplasty. The Laryngoscope.

[19] Redaelli de Zinis LO (2008). Titanium vs hydroxyapatite ossiculoplasty in canal wall down mastoidectomy. Archives of otolaryngology--head & neck surgery.

[20] Truy E, Naiman AN, Pavillon C, Abedipour D, Lina-Granade G, Rabilloud M (2007). Hydroxyapatite versus titanium ossiculoplasty. Otology & neurotology : official publication of the American Otological Society, American Neurotology Society [and] European Academy of Otology and Neurotology.

[21] Schmerber S, Troussier J, Dumas G, Lavieille JP, Nguyen DQ (2006). Hearing results with the titanium ossicular replacement prostheses. European archives of oto-rhino-laryngology.

[22] Yung M, Smith P (2010). Titanium versus nontitanium ossicular prostheses-a randomized controlled study of the medium-term outcome. Otology & neurotology.

[23] Siddiq MA, Raut VV (2007). Early results of titanium ossiculoplasty using the Kurz titanium prosthesis--a UK perspective. The Journal of laryngology and otology.

[24] Jackson CG, Glasscock ME, 3rd, Schwaber MK, Nissen AJ, Christiansen SG, Smith PG (1983). Ossicular chain reconstruction: the TORP and PORP in chronic ear disease. The Laryngoscope.

[25] Brackmann DE (1993). Tympanoplasty with mastoidectomy: canal wall up procedures. The American journal of otology.

[26] Colletti V, Fiorino FG, Sittoni V (1987). Minisculptured ossicle grafts versus implants: long-term results. The American journal of otology.

[27] Krueger WW, Feghali JG, Shelton C, Green JD, Beatty CW, Wilson DF (2002). Preliminary ossiculoplasty results using the Kurz titanium pro- stheses. Otology & neurotology.

[28] Begall K, Zimmermann H (2000). Reconstruction of the ossicular chain with titanium implants. Results of a multicenter study. Laryngorhinootologie.

[29] Murugasu E, Puria S, Roberson JB Jr (2005). Malleus-to-footplate versus malleus-to-stapes-head ossicular reconstruction prostheses: temporal bone pressure gain measurements and clinical audiological data. Otology & neurotology.

[30] Vincent R, Rovers M, Mistry N, Oates J, Sperling N, Grolman W (2011). Ossiculoplasty in intact stapes and malleus patients: a comparison of PORPs versus TORPs with malleus relocation and Silastic banding techniques. Otology & neurotology.

[31] Berenholz LP, Rizer FM, Burkey JM, Schuring AG, Lippy WH (2000). Ossiculoplasty in canal wall down mastoidectomy. Otolaryngology-head and neck surgery.

[32] Quesnel S, Teissier N, Viala P, Couloigner V, Van Den Abbeele T (2010). Long term results of ossiculoplasties with partial and total titanium Vario Kurz prostheses in children. International journal of pediatric otorhinolaryngology.

[33] Moretz WH Jr (1998). Ossiculoplasty with an intact stapes: superstructure versus footplate prosthesis placement. The Laryngoscope.

[34] Woods O, Fata FE, Saliba I (2009). Ossicular reconstruction: incus versus universal titanium prosthesis. Auris, nasus, larynx.

[35] Jaryszak EM, Sampson EM, Antonelli PJ (2009). Biofilm formation by Pseudomonas aeruginosa on ossicular reconstruction prostheses. American journal of otolaryngology.

[36] Wiet RJ, Wiet RM (2010). Experience-driven ossiculoplasty. Operative Techniques in Otolaryn- gology-Head and Neck Surgery.

[37] Mishiro Y, Sakagami M, Adachi O, Kakutani C (2009). Prognostic factors for short-term outcomes after ossiculoplasty using multivariate analysis with logistic regression. Archives of otolaryngology-head & neck surgery.

[38] Shinohara T, Gyo K, Saiki T, Yanagihara N (2000). Ossiculoplasty using hydroxyapatite prostheses: long-term results. Clinical otolaryngology and allied sciences.

[39] Brenski AC, Isaacson B (2009). Reconstruction of the ossicular chain in children. Operative Techniques in Otolaryngology-Head and Neck Surgery.

[40] Pyle GM (2003). Ossicular sculpting for conductive hearing loss. Operative Techniques in Otolaryn- gology-Head and Neck Surgery.

[41] Charlett SD, Scott AR, Richardson H, Hawthorne MR, Banerjee A (2007). Audiometric outcomes of tympanoplasty with hydroxylapatite prosthesis: consultant versus trainees. Otology & neurotology.

